# Health Care Response to CCHF in US Soldier and Nosocomial Transmission to Health Care Providers, Germany, 2009

**DOI:** 10.3201/eid2101.141413

**Published:** 2015-01

**Authors:** Nicholas G. Conger, Kristopher M. Paolino, Erik C. Osborn, Janice M. Rusnak, Stephan Günther, Jane Pool, Pierre E. Rollin, Patrick F. Allan, Jonas Schmidt-Chanasit, Toni Rieger, Mark G. Kortepeter

**Affiliations:** Landstuhl Regional Medical Center, Landstuhl, Germany (N.G. Conger, E.C. Osborn, J. Pool, P.F. Allan);; Walter Reed Army Institute of Research, Silver Spring, Maryland, USA (K.M. Paolino);; Force Health Protection, Fort Detrick, Maryland, USA (J.M. Rusnak);; Bernard Nocht Institute, Hamburg, Germany (S. Günther, J. Schmidt-Chanasit, T. Rieger);; Centers for Disease Control and Prevention, Atlanta, Georgia, USA (P. Rollin);; Uniformed Services University of the Health Sciences, Bethesda, Maryland, USA (M.G. Kortepeter)

**Keywords:** viral hemorrhagic fever, Crimean–Congo hemorrhagic Crimean–Congo hemorrhagic fever virus, fever, ribavirin, infection control, prophylaxis, CCHF, nosocomial infection, viruses, US soldier, health care providers, Germany

## Abstract

In 2009, a lethal case of Crimean–Congo hemorrhagic fever (CCHF), acquired by a US soldier in Afghanistan, was treated at a medical center in Germany and resulted in nosocomial transmission to 2 health care providers (HCPs). After his arrival at the medical center (day 6 of illness) by aeromedical evacuation, the patient required repetitive bronchoscopies to control severe pulmonary hemorrhage and renal and hepatic dialysis for hepatorenal failure. After showing clinical improvement, the patient died suddenly on day 11 of illness from cerebellar tonsil herniation caused by cerebral/cerebellar edema. The 2 infected HCPs were among 16 HCPs who received ribavirin postexposure prophylaxis. The infected HCPs had mild or no CCHF symptoms. Transmission may have occurred during bag-valve-mask ventilation, breaches in personal protective equipment during resuscitations, or bronchoscopies generating infectious aerosols. This case highlights the critical care and infection control challenges presented by severe CCHF cases, including the need for experience with ribavirin treatment and postexposure prophylaxis.

## Abstract 2

Early recognition and implementation of appropriate infection control measures were effective in preventing further transmission.

Crimean–Congo hemorrhagic fever (CCHF) is a life-threatening viral illness endemic to areas of Africa, southeastern Europe, Russia, China, India, and the Middle East. CCHF is caused by infection with a tickborne virus (family *Bunyaviridae*, genus *Nairovirus*), and is generally acquired through the bite of an infected tick or contact with blood or body fluids of infected animals (*1*–*3*). The disease is characterized by the abrupt onset of a febrile illness usually 2–7 d (range 2–14) after exposure to the virus and by subsequent severe changes in mental status, hemorrhagic manifestations, and hepatorenal failure (*1*,*4*). Case-fatality rates vary by region but are 30%–50% (range 1%–73%) in most regions; death generally occurs 5–14 d after symptom onset and is most commonly a result of multi-organ failure, shock, severe anemia, cerebral hemorrhage, and/or pulmonary edema (*1*,*5*).

We report a fatal case of CCHF in a US soldier deployed to Afghanistan, who was aero-evacuated to Germany for treatment, and the documented nosocomial infection of 2 health care providers (HCPs) who were at risk for exposure and had received ribavirin postexposure prophylaxis (PEP). We also review infection control interventions and contact surveillance, both of which were required because of the patient’s severe bleeding and the risk for aerosol production. Research on human subjects was conducted in compliance with US Department of Defense, federal, and state statutes and regulations relating to the protection of human subjects and adheres to the principles identified in the Belmont Report (1979; http://www.hhs.gov/ohrp/humansubjects/guidance/belmont.html).

## The Case

On September 8, 2009, a 22-year-old male US soldier who worked in field operations outside Kandahar City, Afghanistan, sought care at a military medical clinic for a 4-d history of nonbloody diarrhea, abdominal pain, bloody emesis, and fever (39.2°C). The patient reported frequent outdoor activities, tick bites, and exposure to undercooked goat meat and blood the week before the onset of illness. Ciprofloxacin was prescribed for probable gastroenteritis. The patient did not improve by the next day and returned to the clinic, reporting somnolence and lethargy. He was transferred to a Combat Support Hospital at Kandahar Air Base.

Admission laboratory values demonstrated anemia, thrombocytopenia, acute renal insufficiency, and elevated levels of hepatic transaminases. Within a few hours of admission, the patient had worsening lethargy; bloody diarrhea; gingival bleeding; hypoxia requiring intubation; and a hypotensive episode after intubation, which required vasopressor therapy for 24 h. The patient was treated with intravenous levofloxacin (replaced ciprofloxacin), meropenem, fresh-frozen plasma (6 U), platelets (2 U), packed erythrocytes (2 U), and infusions of furosemide and pantoprazole.

On September 10, the patient was emergently aero-evacuated to Landstuhl Regional Medical Center (LRMC; Landstuhl, Germany). During the flight, his respiratory status deteriorated (requiring 100% FiO_2_) and he continued to bleed from multiple sites (nares, gingiva, gastrointestinal, and venipuncture sites). Treatment on route included fresh-frozen plasma (6 U), packed erythrocytes (1 U), and cold-water lavage to decrease upper gastrointestinal bleeding.

The patient arrived at LRMC on September 11 (day 6 of illness); he was in multiorgan, failure and large amounts of bright red blood were in the endotracheal tube. During the initial physical examination, he exhibited respiratory compromise, temperature of 37.3°C, blood pressure of 147/74 mm Hg (reference values for clinical/laboratory values are in Table 1), pulse rate of 118 beats/min, and 90% saturation on 100% oxygen. Significant findings included edematous conjunctivae with mild hemorrhage; nasopharyngeal bleeding; coarse breath sounds; large ecchymoses at venipuncture sites; scattered petechiae on the trunk, arms, and upper thighs; extensive edema of the extremities and scrotum; and melena on rectal examination. Emergent bronchoscopy revealed diffuse bleeding in the airways. Admission laboratory and radiography results (Table 1) supported a presumptive diagnosis of CCHF, and infection control measures for possible viral hemorrhagic fever (VHF) were implemented (*9*).

**Table 1:** Results of laboratory testing and regimen for ribavirin treatment for a US soldier with CCHF, Germany, 2009*

Treatment/test or procedure	Day after symptom onset, date						Reference range
	Day 6, Sep 11	Day 7, Sep 12	Day 8, Sep 13	Day 9, Sep 14	Day 10, Sep 15	Day 11, Sep 16	
Ribavirin treatment†	Oral	IV	IV	IV	IV	None	NA
Test/procedure							
RT-PCR, RNA copies/mL‡	1.2 × 10^9^	ND	6 × 10^9^	ND	3 × 10^8^	ND	NA
Dialysis	ND	ND	Renal	Renal/hep	Renal/hep	Renal	NA
CCHF culture§	+	ND	ND	ND	ND	ND	−
IgM/IgG¶	−/−	ND	−/−	ND	+/+	ND	−
Hemoglobin, g/dL	12.8	7.7	9.1	11.9	9.9	8.5	13.2–17.1
Hematocrit, %	35.3	21.6	25.4	33.5	27.7	23.7	38–50
Leukocyte count, × 10^3^/μL	9.6	8.8	4.9	4.0	3.4	3.5	3.5–10.5
Platelets, × 10^3^/μL	14,000	68,000	62,000	93,000	126,000	77,000	151–356
Creatinine, mg/dL	7.8	8.7	5.1	2.7	1.4	0.9	0.8–1.5
BUN, mg/dL	67	72	32	8	2	<2	8–26
Sodium, mmol/L	140	146	143	142	141	147	137–145
Potassium, mmol/L	4.7	5.2	4.0	5.0	4.3	3.4	3.6–5.1
Bicarbonate, mmol/L	20	12	19	18	28	33	22–31
Chloride, mmol/L	110	102	98	103	100	101	101–111
Lactate, mmol/L	3.0	14.9	17.8	8.7	7.7	7.4	0.7–2.1
Glucose, mg/dL	92	187	93	68	82	89	74–106
AST, U/L	1,472	3,957	11,295	9,628	9,061	5,967	15–41
ALT, U/L	411	1,838	2,854	2,151	1,805	1,122	17–63
LDH, IU/L	756	ND	ND	ND	ND	ND	98–192
Alkaline phos, UL	186	123	163	202	254	354	38–126
Bilirubin							
Total, mg/dL	1.8	5.8	6.7	8.1	9.2	10.4	0.2–1.3
Direct, mg/dL	1.1	ND	3.3	3.2	3.0	3.0	0.1–0.3
aPTT, s	106.9	89.8	56.6	59.3	67.4	52.7	28.2–40.3
Prothrombin time, s	21.8	22.4	22.3	14.9	19.7	24	11.9–15.1
Fibrinogen, mg/dL	143	190	238	156	153	111	168–407
D-dimer, µg/dL	20	ND	ND	ND	ND	ND	<5
Albumin, g/dL	2.8	2.8	3.7	4.2	4.7	4.8	3.5–5.0
CPK, U/L	1,437	1,528	1,889	3,008	4,728	4,973	55–170
Myoglobin, ng/mL	1,226.5	ND	ND	ND	ND	ND	17.4–105.7
Other							
Malaria smear	−**	**	**	**	**	**	NA
Bacterial cultures#	−**	**	**	**	**	**	NA
Radiology							
X-ray/CT, chest	ND	Moderate to severe pulmonary edema and atelectesis	ND	ND	ND	ND	NA
CT, abdomen	ND	Ascites, gallbladder edema	ND	ND	ND	ND	NA
Cytokines							
Interleukin							
10, pg/mL ††	515	ND	1,498	ND	904	ND	<7
6, pg/mL ††	1,530	ND	>3,023	ND	2,439	ND	<15
IFN-γ, pg/mL ††	59	ND	390	ND	125	ND	<15
TNF-α, pg/mL ††	77	ND	56	ND	100	ND	<15
Growth factors							
PLGF, pg/mL ††	203	ND	64	ND	81	ND	<25
sVEGF-R1, pg/mL ††	2,930	ND	13,903	ND	13,308	ND	<180

*aPTT, activated partial thromboplastin time; ALT, alanine aminotransferase; AST, aspartate aminotransferase; BUN, blood urea nitrogen; CCHF, Crimean–Congo hemorrhagic fever; CPK, creatine phosphokinase; CT, computerized tomography; hep, hepatic; IFN-**γ,** interferon γ; IV, intravenously; LDH, lactate dehydrogenase; NA, not applicable; ND, not determined; phos, phosphatase; PLGF, placental growth factor; RT-PCR, reverse transcription PCR; sVEGF-R1, soluble vascular endothelial growth factor receptor 1; TNF-α, tumor necrosis factor α; −, negative; +, positive. †On day 6, an initial 4-g loading dose (LD) of oral ribavirin was administered via nasogastric tube, followed by 1,200 mg 6 h later. On day 7, a partial LD of 22 mg/kg was administered IV (because of 60% bioavailability of oral ribavirin and poor absorption with gastrointestinal bleeding) followed by 16-mg/kg doses every 6 h (per dose-reduction protocol). Beginning day 9, 14 mg/kg was administered every 6 h, with an extension of the dosing interval to every 8 h on day 10 because of severe renal failure (only a minimal amount of drug is removed through dialysis) (*3*). ‡Real-time RT-PCR for virus quantification and *Nairovirus* spp.–specific gel-based RT-PCR coupled with PCR product sequencing to confirm the diagnosis (*6*,*7*). §CCHF culture of blood and urine (virus was isolated on Vero cells and sequenced) (*8*). ¶CCHF-specific IgM/IgG by indirect immunofluorescence assay using CCHF virus–infected cells; assay performed at Bernard Nocht Institute, Hamburg, Germany. #Culture of blood, urine, and sputum samples. **Malaria smear and culture results were not specifically obtained on day 6; multiple cultures were performed. ††Testing for cytokines and vascular endothelial growth factors and their soluble receptors of blood were performed in the Biosafety Level 4 facility of Bernard Nocht Institute by using Quantikine Immunoassays (R&D Systems Europe, Abingdon, UK), according to the manufacturer’s instructions.

On day 7 of illness, reverse transcription PCR (RT-PCR) of a serum sample obtained at admission showed a CCHF viral load of 1.2 × 10^9^ copies/mL (CCHF virus was later isolated from blood and urine samples obtained on day 6 of illness; CCHF IgM and IgG serologic results were negative on day 6 of illness) (Table 1). Oral and then intravenous ribavirin therapy were initiated; the intravenous ribavirin was administered within 24 h of admission and under an Investigational New Drug protocol after the patient’s family gave consent (Table 1) (*3*,*10*). Treatment with the broad-spectrum antibiotics was discontinued. Repetitive emergency bronchoscopy was required to control severe pulmonary hemorrhage.

On day 8 of illness, the patient’s pulmonary status continued to deteriorate: development of adult respiratory distress syndrome required placement of bilateral chest tubes to drain bloody pleural effusions, administration of nitric oxide, and use of advanced inverse ratio bilevel mechanical ventilation. The severe adult respiratory distress syndrome was complicated by massive pulmonary hemorrhaging that required multiple bronchoscopies and infusion of blood products (packed erythrocytes [32 U], fresh frozen plasma [80 U], platelets [34 packs], factor VII [4 U], and cryoprecipitate [3 U]). Tris (hydroxymethyl) amino methane and continuous hemodialysis were initiated for progressive renal failure and severe acidosis (serum creatinine 8.2 mg/dL, bicarbonate 12 mmol/L), and multiple boluses of glucose were given for recurrent hypoglycemia.

On day 9 of illness, the patient’s oxygenation and blood product transfusion requirements lessened, but hypoglycemia and acidosis persisted; a bicarbonate drip was initiated. Because fulminant hepatic failure occurred (aspartate aminotransferase 9,628 U/L, alanine aminotransferase 2,151 U/L, total bilirubin 8.1 mg/dL), liver dialysis was initiated by using a liver albumin dialysis machine. Early on day 11 of illness, liver dialysis was discontinued because the patient's condition appeared to be stabilizing (serum viral load and hepatic transaminases were decreasing, and less ventilatory support and fewer blood product transfusions were required). However, neurologic examination later that morning showed bilateral fixed and dilated pupils, and before brain imaging was possible, the patient suffered a cardiorespiratory arrest. A postmortem computer tomographic scan image demonstrated diffuse cerebral and cerebellar edema, a small right frontal parenchymal hemorrhage, and bilateral cerebellar tonsil herniation.

## Hospital Infection Control

Because a CCHF diagnosis was not considered likely at the time of the initial 2 emergency bronchoscopies, standard precautions for infection control were used. The 2 bronchoscopists wore a gown, gloves, eye protection, and surgical masks; other persons in the room wore N95 respirators or surgical masks. More stringent infection control measures for VHFs, including airborne precautions, were implemented once a diagnosis of CCHF was considered (*5*) (Table 2). The patient was placed in an airborne-infection isolation room with an anteroom, which had restricted visitation and intensive care unit (ICU) entry. Sign-in sheets tracked who entered the patient’s room; those entering were required to wear a fluid-resistant gown, gloves, N95 respirator, eye protection/face shield, and shoe coverings. Biohazard suits (Tyvek; DuPont, Richmond VA, USA) with powered air-purifying respirators were worn during subsequent bronchoscopies and chest tube placement. Infection prevention and control staff provided refresher training on the proper donning and doffing of personal protective equipment (PPE) and oversaw the decontamination of bronchoscopes and ventilators. The procedures for disposing of medical wastes were in accordance with German regulations for handling infectious biohazardous materials (Table 2).

**Table 2:** Isolation, infection control, PPE, and decontamination procedures used by a health care center during treatment of a US soldier infected with Crimean–Congo hemorrhagic fever virus, Germany, 2009*

Focus	Procedure
Patient	Placed in AIIR with an anteroom. Restricted visitation; sign-in sheet to track personnel entry. Entry required wearing of fluid-resistant gown and gloves (gloves pulled over edge of gown sleeve cuff), fit-tested N95 respirator, eye protection/face shield, and shoe coverings; disposal of PPE in anteroom before exiting. IPaC staff performed hands-on refresher training sessions for proper donning and doffing of PPE and for respiratory procedures (i.e., suctioning). Biohazard suits with PAPRs worn during bronchoscopies and chest tube placement.
Ventilator	Labeling of ventilators used on patient; IPaC staff–observed cleaning to ensure proper decontamination/terminal disinfection before use on another patient. Bleach 10% solution used to clean ventilators; bellows replaced; circuits discarded; internal removable parts were removed, bagged, and sterilized (viral desiccation).
Bronchoscope	Two dedicated bronchoscopes, equipment, and bronchoscope tower (labeled restricted use); IPaC staff–observed cleaning to ensure proper decontamination/terminal disinfection. Cleaning/decontamination of endoscope performed after each procedure: endoscope soaked in enzymatic detergent to remove soils (to reduce risk of splashing, no scrubbing performed); decontaminated endoscope placed in AER with a biologic indicator testing (to ensure proper decontamination/cleaning); each endoscope load underwent 2 cycles in the AER before reuse.
Medical waste	All medical waste placed in RMW bags located inside patient’s room, sprayed, and then placed in a rigid plastic container (labeled biohazard/RMW) before disposal and incineration, following Germany’s regulations for handling infectious biohazardous wastes. Disposable sharps placed in sharps containers, autoclaved, and contained in protected location until disposal/decontamination (incineration). Suctioned containers holding blood-contaminated fluids, oral and respiratory secretions, bronchoscopy drainage fluids, or other drainage from patient snapped closed and contained/stored/labeled as biohazard/RMW before disposal/incineration.
Linen	All linen (disposable isolation gowns of HCWs, sheets and gowns of patient) placed in labeled regulated medical waste bags and sealed. When full, these RMW bags were then stored in larger (50 gallon) RMW containers and secured in a RMW holding area (another AIIR in the ICU that was labeled and secured as a RMW holding area) until transport for incineration. The outside of all RMW bags/containers wiped down with a 10% bleach solution before transport.
Medical laboratory	Phlebotomy/laboratory tests limited to most critical samples; performed by a single laboratory technician. All specimens placed in a plastic zip-locking bag that was placed inside a rigid plastic container and then inside a second similar (but larger) plastic container with lid taped to the container (biohazard/RMW labels). All specimens directly transported to the laboratory. Except for blood and urine samples for diagnostic tests, specimens not pretreated with polyethylene glycol p-tert-octylphenyl ether under a laminar flow hood to reduce viral load before shipment to Bernard Nocht Institute because of concern treatment may interfere with validity of laboratory tests (but will be recommended in future cases). PPE for laboratory workers included gown, gloves, and N95 respirators (N95 respirators worn because specimens with a high viral load were tested in analyzers outside the BSC). Centrifugation of specimens performed within a Class II BSC. Chemical disinfection of instruments/equipment performed immediately after each use with 10% bleach solution (or per manufacturer’s recommendation). All specimens and nonreusable equipment autoclaved before disposal, then incinerated per Germany’s regulatory requirements.
Terminal decontamination	Bleach 10% solution or standard hospital-grade disinfectants used for terminal cleaning of all surfaces and equipment, of patient’s room, and of aero-evacuation airplane. Terminal cleaning of ICU room overseen by IPaC staff.
Cadaver	Body sprayed with 10% bleach, placed in a body bag that was then decontaminated with 10% bleach solution, and then in a second sealed body bag that was decontaminated with 10% bleach solution before transfer to morgue. Embalming performed (generally not recommended due to exposure risk) by personnel wearing biohazard suits with hood, full face respirators, and double gloves overlapping sleeves of biohazard suit (duct-taped at wrists). Embalming procedures observed by IPaC staff. Body maintained in room at 1.1°–3.3°C. Daily RT-PCR of serum samples and RT-PCR of deep tissue samples on days 1 and 6 after embalming (confirmed negative). Chemical disinfection of nonsurgical instruments and equipment; surgical instruments also sterilized. Terminal decontamination of room.
*AER, automatic endoscope reprocessor; AIIR, airborne-infection isolation room; BSC, Class II biosafety cabinet; HCWs, health care workers; ICU, intensive care unit; IPaC, infection prevention and control; PAPRs, powered air-purifying respirators; PPE, personal protective equipment; RMW, regulated medical waste; RT-PCR, reverse transcription PCR.	

Laboratory interventions involved limiting blood draws and analyses to the most critical samples and to a single laboratory technician. Laboratory personnel wore gowns, gloves, and N95 respirators. Centrifugation of specimens was performed within a Class II biosafety cabinet. Laboratory equipment was decontaminated immediately after use and all nonreusable equipment was autoclaved before disposal. Blood and urine samples were pretreated with polyethylene glycol (to reduce viral load) before being shipped to Bernard Nocht Institute (Hamburg, Germany) for CCHF diagnostic testing. The cadaver was placed in 2 sealed body bags; the outside of each bag was decontaminated with a 10% bleach solution. RT-PCR analysis of deep cadaver tissue samples was performed after embalming and confirmed to be negative. Bleach (10%) or standard hospital-grade disinfectants were used for terminal cleaning of the patient’s room and all surfaces and equipment in the airplane used to aero-evacuate the patient to Germany.

## Outbreak Investigation

Contact tracing commenced immediately after diagnosis and included a wide group of persons who may have been at risk for exposure to the patient’s blood/body fluids: personnel in the patient’s deployed unit, persons at the Combat Support Hospital in Kandahar, the medical evacuation team, and persons at LRMC (HCPs, laboratory workers, and transport, housekeeping, and volunteer staff). Among these contacts, 18 HCPs were identified as having been at risk for exposure and were offered oral ribavirin PEP (off-label use); 16 of the 18 accepted treatment (Table 3). Most of the 18 HCPs were present during bronchoscopies or ventilation procedures that used a bag-valve-mask device and had reported blood splashes on their gowns. Although there were no known percutaneous exposures, 2 HCPs reported blood on intact skin. Also, some HCPs wore only a surgical mask as PPE during the initial bronchoscopies and/or were unsure if they had always maintained a properly fitted N95 respirator during subsequent bronchoscopies. The group of 16 HCPs who accepted ribavirin PEP included a medic in Kandahar who had a blood exposure on his ungloved hand during an emergency intravenous catheter insertion and a physician in Kandahar who emergently intubated the patient without wearing an N95 respirator. The group also included an LRMC ICU nurse and respiratory therapist, both of whom had met the patient on arrival at LRMC and manually ventilated him during transport to the ICU without wearing a mask or eye protection; during the transport, the patient was actively bleeding from intravenous catheter sites and coughing blood into the endotracheal tube. These 2 HCPs (and others) were also present during the initial 2 bronchoscopies, during which they may not have worn surgical masks at all times (and no eye protection) and their gowns had been soaked from blood exposures. In addition, the respiratory therapist’s face shield dislodged immediately after being sprayed with blood while she was manually ventilating the patient using a bag-valve-mask device during a life-threatening hypoxic event. The ICU nurse also had blood contact on her skin (wrist) during resuscitation, when her gown sleeve slipped from the glove. The respiratory therapist and ICU nurse were also among the HCPs, aside from a few physicians, who spent the most time directly caring for the patient. The remaining 72 personnel had unlikely/no identifiable exposure risk and were instructed to have their temperatures taken twice daily for 15 d and to contact the infectious diseases physician for any febrile illness within this same time period (Table 3).

**Table 3:** Surveillance criteria and PEP, by exposure risk, for contacts of US soldier with fatal Crimean–Congo hemorrhagic fever, Germany, 2009*

Group no.	No persons	Risk	PEP and monitoring
1	18	Contact of skin or mucous membranes with contaminated blood or body fluids; present during bronchoscopy or during use of bag-valve-mask ventilation device (risk of aerosolization of infectious blood/body fluids likely) and without proper PPE†	Oral ribavirin PEP offered; baseline and at least weekly chemistries and CBC; CCHF acute/convalescentphase titers‡; monitoring for fever (twice daily) and for CCHF symptoms and medication side effects (for 15 d in clinic)
2	31	Present during bronchoscopy or during use of bag-valve-mask ventilation device (even with proper PPE)†; known contact with contaminated blood or body fluids but wore proper PPE and without PPE breaches† (no known mucosal or skin contact with infectious blood/body fluids); laboratory workers who performed tests on specimens (removed specimens from container) and wore proper PPE†	Monitoring for fever twice daily for 15 d (in clinic); self-observation and reporting of signs or symptoms e.g., fever) for 15 d
3	41	Persons in patient’s room who wore proper PPE and without PPE breaches and no contact with infectious blood/body fluids†; laboratory workers who handled laboratory specimens (but did not remove specimens from container) and wore proper PPE†	No active monitoring; self-observation and reporting of signs or symptoms (e.g., fever) for 15 d

*CBC, complete blood count; CCHF, Crimean–Congo hemorrhagic fever; PEP, postexposure prophylaxis; PPE, personal protective equipment. †Proper PPE for aerosol exposure included gown, gloves, N95 respirator, and protective eyewear; powered air-purifying respirators and full biohazard suits were required during bronchoscopies and chest tube placements by physician performing the procedure. ‡ELISA for CCHF-specific IgM and IgG performed at the Centers for Disease Control and Prevention, Atlanta, Georgia, USA (*11*).

An oral ribavirin PEP regimen of 600 mg twice daily for 7 d was recommended initially; this dosage was based on drug availability, drug tolerance, and dosage regimens reported in the literature (*3*). Seventy-two hours later, more oral ribavirin became available, and the 16 HCPs were offered a 4-times-daily dosing regimen (600 mg/dose) and/or extension of PEP from 7 to 14 d. Because of the drug’s side effects, all HCPs chose to remain on a twice-daily dosing regimen; only 2 HCPs accepted an extension of PEP to 14 d. Side effects (mainly fatigue, dyspepsia, nausea, and headache) were reported by all 16 HCPs. Of the 12 HCPs compliant with blood draws, 10 showed an increase in total bilirubin (range 1.2–5.7 mg/dL) and 2 had mild anemia (nadir hemoglobin 11.9 g) attributed to hemolysis caused by ribavirin. Leukopenia was observed in 1 HCP (leukocyte count 2,800 cells/mm^3^). 

To assess possible seroconversion in the patient’s contacts, initial and follow-up (4–6 wk) blood samples were obtained from personnel in the patient’s deployment unit (n = 62), persons at the Combat Support Hospital in Kandahar (n = 55), and persons at LRMC (n = 74) and sent for serologic testing at the Centers for Disease Control and Prevention (Atlanta, GA, USA) (*11*). Although baseline serologic testing was not done, results of serologic testing done at 8 weeks for 2 HCPs who received oral ribavirin PEP (the ICU nurse and the respiratory therapist at LRMC) were consistent with acute CCHF seroconversion: CCHF virus–specific IgM and IgG titers were >6,400, and over the next 2 mo, IgM titers declined (Table 3). These 2 HCPs were the most symptomatic of the 16 persons who received ribavirin PEP, and the only persons to seek medical attention for their symptoms. Symptoms were initially noted 4–5 d after exposure (day 4 of ribavirin PEP). The ICU nurse had moderate abdominal discomfort and jaundice; a total bilirubin of 5.7 mg/dL was the only abnormal laboratory value (direct bilirubin was 0.2 mg/dL). The respiratory therapist experienced fatigue, myalgias, and chills (no documented fever). She had a reference bilirubin level and mild leukopenia (leukocytes decreased from 4,200 cells/mm^3^ to 2,800 cells/mm^3^), and she missed 3 d of work because of her symptoms. For both HCPs, the symptoms and laboratory abnormalities were initially attributed to side effects of ribavirin therapy (particularly the elevated bilirubin level and gastrointestinal discomfort); in retrospect, the respiratory therapist’s symptoms most likely represented CCHF symptoms ameliorated by ribavirin PEP. Ameliorated CCHF as a cause of symptoms in the ICU nurse could not be excluded.

## Discussion

This fatal case of CCHF in a US soldier illustrates several issues regarding clinical management, infection control measures, epidemiologic investigation, and ribavirin PEP in CCHF infection. Early recognition and diagnosis of CCHF is paramount, so that medical care and appropriate infection control measures can be implemented in the initial phase of illness and, thereby, improve survival and prevent nosocomial transmission. Delayed diagnosis and implementation of infection control measures can result in the need for extensive public health resources to evaluate and follow up on exposed HCPs and contacts (*12*–*17*).

The greatest risk for nosocomial transmission of the CCHF virus has been from percutaneous exposure with contaminated needles. Blood exposure on intact skin is a much lower risk, but sporadic transmission has been reported after skin or mucosal exposures to infectious blood. Nosocomial infection has also been reported without a clear source of virus transmission, and possible droplet (patient-to-patient) transmission has been reported (*3*).

Bronchoscopies and other procedures producing infectious aerosols were potential sources of CCHF virus transmission to the 2 HCPs reported here; however, no other HCPs who were present during the initial 2 bronchoscopies showed seroconversion. Thus, it is probable that virus transmission resulted from exposure to infectious blood during initial transport of the patient to the ICU, when neither HCP wore proper PPE (surgical mask/N95 respirator) while manually ventilating the patient or from a breach in PPE (particularly, for 1 HCP when her face shield dislodged during a resuscitation procedure). Of concern, both HCPs were unaware of their PPE breaches/exposures during resuscitation efforts; they were noted by other HCPs. The PPE breaches and potential blood/body fluid exposure risks led to the use of biohazard suits and powered air-purifying respirators during subsequent bronchoscopies and chest-tube placements. Compared with face shields, powered air-purifying respirators are less likely to become dislodged. The 2005 Centers for Disease Control and Prevention’s modified PPE guidelines for suspected VHFs at US hospitals note that extenuating circumstances (i.e., procedures generating aerosols, severe pulmonary involvement, or copious bleeding) may necessitate an increase in PPE (i.e., plastic aprons, leg/shoe coverings) or airborne precautions (*9*).

The processing of serum specimens with high viral loads in analyzers outside the Class II biosafety cabinet in a non–negative-pressure laboratory was a concern. Pretreatment of specimens to reduce viral load (i.e., with heat inactivation or polyethylene glycol) will be recommended in future cases, even though such treatment may affect laboratory results (*9*,*18*). Also, the CCHF virus has been detected in urine by RT-PCR as late as day 36 after illness onset; however, infectivity has been unclear because the virus had not been previously cultured from urine (*19*,*20*). The culture of the CCHF virus in patient samples obtained on day 6 of illness indicates that a positive RT-PCR result for urine may represent viable virus and the potential for late transmission of virus by patients who have recovered from CCHF.

Ribavirin, a synthetic purine nucleoside analog, has demonstrated in vitro activity against CCHF virus and decreased death rates in infected suckling mice (*3*). Ribavirin efficacy in humans has not been evaluated in placebo-controlled trials against CCHF because of ethical concerns. However, retrospective analyses of ribavirin-treated CHHF virus–infected cohorts (compared with untreated historical controls) often report a decrease in CCHF-associated death if given within 72 h after the onset of symptoms (*3*). Anecdotal reports of ribavirin PEP in CCHF cases are limited, but they also suggest a possible benefit in preventing or ameliorating disease in most HCPs (*2*,*3*). However, the optimal dosage and duration of oral ribavirin for CCHF prophylaxis is unknown. PEP regimens in the literature range from 200 mg twice daily to as high as 4 g daily for 5–14 d; the most common regimen is 500 mg 3–4 times/d for 7-10 d. A 2-g loading dose is recommended in some regimens, particularly when treatment initiation is delayed (*3*,*21*). Drug side effects (mainly gastrointestinal intolerance and fatigue) often limit the dosage and duration of ribavirin PEP (*3*).

CCHF virus IgM and IgG titers in the 2 seropositive HCPs corresponded to acute infection from nosocomial transmission because these persons had no other risk factors for recent exposure to the virus. The mild/absent CCHF symptoms in these 2 HCPS who received a lower dose and duration of ribavirin PEP (600 mg twice daily for 7 d) may provide further insight regarding the potential benefit and dosage regimen for oral ribavirin PEP. The estimation of 88% of CCHF cases in Turkey being subclinical in a recent seroprevalence study would likely necessitate a controlled clinical trial to assess the efficacy and dose for ribavirin PEP (*22*).

On arrival at LRMC (day 6 of illness), the patient had a poor prognosis for survival: ribavirin treatment had been delayed >4 d after symptom onset; he was somnolent; and he had severe bleeding and coagulopathy, significantly elevated levels of hepatic transaminases, a platelet count of <20,000/mm^3^, and a serum viral load of >1 × 10^8^ copies/mL (*4*,*23*–*28*). However, with supportive care, the patient showed clinical improvement (i.e., improved respiratory status, decreased bleeding and blood product requirements, and improved end-organ function). Continuous renal replacement therapy was particularly helpful in controlling the patient’s life-threatening metabolic derangements; hepatic replacement therapy was of uncertain benefit and interfered with the optimal use of continuous renal replacement therapy. On day 10 of illness, the patient was able to follow commands, his serum viral load was declining, and CCHF-specific IgG was present.

There were multiple possible reasons for the fatal brain herniation on day 11 of illness. CCHF infection can cause endothelial cell dysfunction (with increased vascular permeability) through the induction of cytokines (tumor necrosis factor-α, interleukin-6, interleukin-10, interferon [IFN]-**γ)**, which can result in cerebral edema (*28*,*29*). Increase in these cytokines and markers for increased endothelial cell permeability (i.e., vascular endothelial growth factor [VEGF]-A and soluble VEGF receptor 1) have been correlated with increased serum viral load and increased risk of death and/or severe CCHF disease (Table 2) (*30*–*36*). In a similar VHF case (Marburg virus disease) with elevated levels of cytokine and soluble VEGF receptor 1 in which brain herniation occurred, the patient had cerebral edema that was not controlled with renal and hepatic dialysis, mannitol, and hypotonic saline (*37*). Other contributing factors for cerebral edema include possible direct effects of the virus in the brain and the combination of hepatic failure, persistent acidosis, and large osmotic shifts caused by dialysis; frontal lobe hemorrhage was not a significant factor because of its small size and location.

Severe CCHF has been attributed to a delayed IFN-α response in infected persons and to insensitivity of infected cells to the effects of the response (i.e., down-regulation of the host’s innate immune response) (*38*,*39*). The failure of ribavirin to prevent death in mice lacking type I IFN-α receptors (serum viral load was reduced, only delaying death), its poor ability to cross the blood–brain barrier, and its delayed initiation in this soldier, suggest a minimized effect of ribavirin against CCHF virus in this fatal case (*3*,*40*). However, a newer antiviral drug, favipiravir (a nucleoside analog also known as T-705), may offer promise as a future treatment option for CCHF. Mice lacking type I IFN-α receptors had no detectable CCHF-specific antibody if given favipiravir within 2 d of CCHF virus challenge, and all mice treated within 3 d of challenge survived with no detectable virus in the blood or organs (*40*).

This case of a soldier who died from CCHF illustrates the need to maintain an index of suspicion for CCHF and other VHFs in febrile travelers returning from VHF-endemic areas so that supportive care and appropriate infection control measures can be implemented early in the course of illness. This case highlights the critical care challenges in caring for a patient with severe CCHF and describes nosocomial CCHF virus infection in 2 HCPs who were receiving oral ribavirin PEP (600 mg twice daily). The 2 nosocomial infections stress the need for infection control policies that educate HCPs to use contact and droplet precautions (minimal requirements) when caring for patients presenting with fever and hemorrhage. In tertiary-care medical settings, procedures and emergency resuscitations performed on VHF patients with severe hemorrhage may pose risks for HCPs that are different from those in smaller hospitals in developing countries, where such procedures may not be available. Because the risk for aerosol production, splashing blood, and breaches in PPE (i.e., dislodged face shields, face masks, sleeve separation from gloves) is highest during resuscitative efforts, in this VHF case with severe hemorrhage, Tyvek suits with powered air-purifying respirators were indicated for the HCPs at highest risk for possible exposure to infectious materials (i.e., bronchoscopists). Although in this case, the potential antiviral effect of ribavirin may have been decreased because of late initiation of the drug and poor penetration of the blood–brain barrier, ribavirin may have contributed (along with CCHF IgG) to the patient’s improved clinical condition and decreased serum viral load. In addition, the probable CCHF virus seroconversion of 2 HCPs who had ameliorated or no symptoms after receiving ribavirin PEP may contribute further to the experience with and dosing regimen for ribavirin PEP in HCPs exposed to CCHF virus. 
